# Probing the geometry of copper and silver adatoms on magnetite: quantitative experiment *versus* theory†
†Electronic supplementary information (ESI) available: Experimental and computational details, as well as further details on the results and analyses. See DOI: 10.1039/c7nr07319d


**DOI:** 10.1039/c7nr07319d

**Published:** 2018-01-15

**Authors:** Matthias Meier, Zdeněk Jakub, Jan Balajka, Jan Hulva, Roland Bliem, Pardeep K. Thakur, Tien-Lin Lee, Cesare Franchini, Michael Schmid, Ulrike Diebold, Francesco Allegretti, David A. Duncan, Gareth S. Parkinson

**Affiliations:** a University of Vienna , Faculty of Physics and Center for Computational Materials Science , 1090 Vienna , Austria; b Institute of Applied Physics , TU Wien , 1040 Vienna , Austria; c Diamond Light Source , Harwell Science and Innovation Campus , Didcot , OX11 0QX UK . Email: david.duncan@diamond.ac.uk; d Physics Department E20 , Technical University of Munich , 85748 Garching , Germany

## Abstract

Accurately modelling the structure of a catalyst is a fundamental prerequisite for correctly predicting reaction pathways, but a lack of clear experimental benchmarks makes it difficult to determine the optimal theoretical approach. Here, we utilize the normal incidence X-ray standing wave (NIXSW) technique to precisely determine the three dimensional geometry of Ag_1_ and Cu_1_ adatoms on Fe_3_O_4_(001). Both adatoms occupy bulk-continuation cation sites, but with a markedly different height above the surface (0.43 ± 0.03 Å (Cu_1_) and 0.96 ± 0.03 Å (Ag_1_)). HSE-based calculations accurately predict the experimental geometry, but the more common PBE + U and PBEsol + U approaches perform poorly.

## Abstract 2

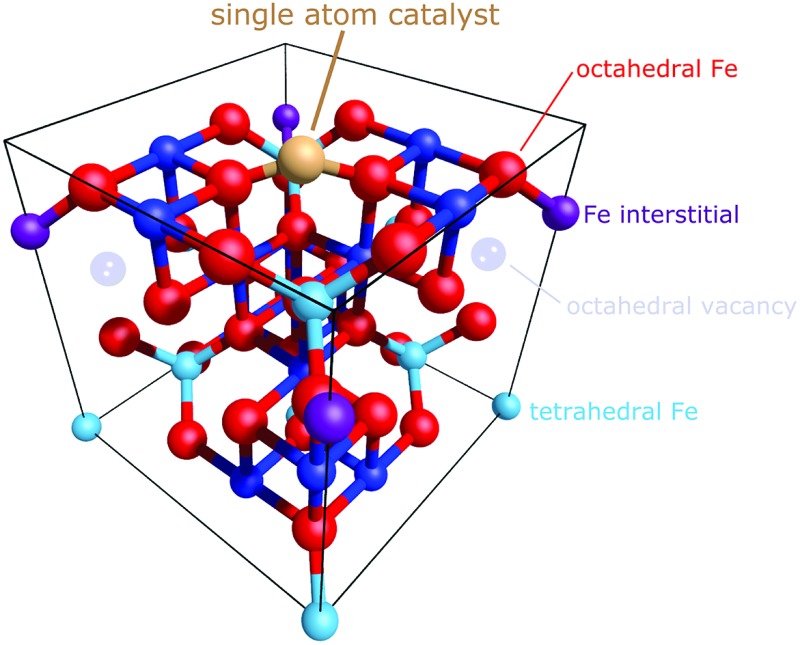
Benchmarking DFT calculations against precise normal incidence X-ray standing wave measurements.

## 

Density functional theory (DFT) has become an indispensable tool in modern catalysis research, allowing us to understand long-observed trends in reactivity and unravel complex reaction mechanisms.^1^ Rapid advances in computational power have fueled efforts to screen, and even predict catalysts from first principles,^2^–^4^ but real predictive power requires adsorption energies and reaction barriers to be quantitatively correct. This must begin with an accurate description of the catalyst, but there is little in the way of solid experimental benchmarks^5^ to test the different exchange–correlation functionals,^6^–^11^ van der Waals corrections, both (semi-) empirical^12^–^14^ and non-empirical,^15^,^16^ and methods beyond DFT.^17^–^23^

The emerging field of single-atom catalysis (SAC)^24^–^30^ is a case in point. Although, there are reports of highly-active single-atom catalysts,^31^–^37^ the field remains controversial^38^ because such systems are difficult to characterize experimentally. Moreover, the catalytic mechanism is often proposed on the basis of theoretical calculations,^31^,^36^,^39^–^43^ which utilize an idealized catalyst support with metal adatoms adsorbed at high-symmetry sites on a low index facet. Thus, the 1 : 1 equivalence of experimental and theoretical data is difficult to establish.

FeO_*x*_ nanocrystallites have been observed as a support material that can anchor single adatoms, with both Pt and Ir adatoms exhibiting catalytic activity.^31^,^33^ These two studies utilized the same coprecipitation method to generate the nanocrystallites and, in the work of Lin *et al.*,^33^ were observed by X-ray diffraction to be primarily magnetite crystallites. Recently we discovered that the (001) surface of a magnetite single crystal can stabilize ordered arrays of metal adatoms (*e.g.* Au,^44^ Pd,^45^ and Pt^46^).^47^ These adatoms were found to be homogenously distributed up to a comparatively high coverage and with high thermal stability, and is therefore a promising model system to provide insight into single atom catalysts supported on FeO_*x*_ nanocrystallites. It is this remarkable density, stability, and homogeneity of adatom arrays that offers the opportunity to perform a precise structural determination, and test the ability of DFT-based calculations to accurately model these dispersed lone adatoms. To that end, we report a normal incidence X-ray standing waves (NIXSW^48^) study of two members of this family: Ag_1_ and Cu_1_ adatoms on Fe_3_O_4_(001). These adatoms were chosen for their nobility, in order to avoid undesired adsorption of the residual gases found in ultra-high vacuum, and thus are used as a comparatively simple benchmark with which to test the performance of theoretical calculations. Both metals were determined to adsorb in a surface tetrahedral cation site, with significantly different adsorption heights (0.96 ± 0.03 Å for Ag and 0.43 ± 0.03 Å for Cu). DFT calculations using the Heyd–Scuseria–Ernzerhof (HSE) functional^23^ reproduce the geometry well, but the more common Perdew–Burke–Ernzerhof + U (PBE + U) and PBEsol + U approaches perform poorly. Although improved structural agreement can be achieved by constraining the lattice parameter to the experimental value, the failure to meet this experimental benchmark raises concern over this widely used functional in the field of adatoms on metal oxide surfaces.

Full experimental details can be found in the ESI.† The as-prepared surface exhibits a sharp (√2 × √2)R45° low energy electron diffraction (LEED) pattern (not shown), and scanning tunneling microscopy (STM) images reveal rows of surface Fe_oct_ atoms running in the [110] directions (see Fig. 1A and B). Here, Fe_oct_ refers to atoms with octahedral coordination to oxygen in bulk Fe_3_O_4_. The surface Fe_oct_ rows exhibit a characteristic distortion due to an ordered array of subsurface cation vacancies and interstitials,^47^ the so-called subsurface cation vacancy (SCV) reconstruction.

**Fig. 1:**
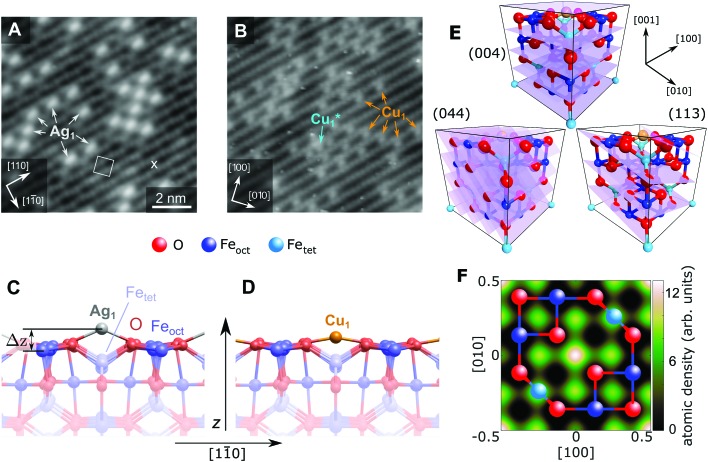
STM image of (A) 0.27 ML Ag and (B) 0.41 ML Cu on the Fe_3_O_4_(001) surface (*V*_sample_ = +1.2 V/+2.0 V, respectively, *I*_tunnel_ = 0.3 nA). All Ag_1_ adatoms occupy the “narrow” site marked by an × in the figure and the (√2 × √2)R45° unit cell is indicated by a white square. The stable majority site (Cu_1_) and metastable minority site (Cu_1_*) are labelled. (C, D) Side view of the optimum Ag/Fe_3_O_4_(001) and Cu/Fe_3_O_4_(001) structures determined by HSE, with a height above the relaxed Fe_oct_ surface atoms (Δ*z*) of 1.12 Å and 0.59 Å, respectively. (E) Unit cell of Fe_3_O_4_(001) with the experimentally determined site of the Cu_1_ adatom. The (004), (113) and (044) planes utilized in the NIXSW experiments are indicated. (F) A 2D atomic density map of the Cu adatom obtained from the NIXSW measurements, as described in the ESI,† overlaid with a ball-and-stick model representing an idealized Fe_oct_O_2_ bulk termination. The adsorption site can be clearly identified at the center and corners of the map, corresponding to an oxygen bridge site.

Fig. 1 shows STM images of the surface following the deposition of 0.27 ML Ag (a) and 0.41 ML Cu (b). Isolated adatoms appear as bright protrusions between the surface Fe_oct_ rows, with apparent heights relative to the surface Fe_oct_ rows of 1.1 ± 0.3 Å and 0.6 ± 0.2 Å for Ag_1_ and Cu_1_ respectively. As observed previously,^49^ adatom adsorption occurs almost exclusively at the site marked by an × in Fig. 1A, *i.e.*, where the separation of the Fe_oct_ rows appears narrowest with a sample bias of 1–1.5 V.^47^ Approximately 10% of the Cu adatoms occupy an alternative adsorption site, Cu_1_*, after room temperature deposition. This site is metastable, and can be converted into regular Cu_1_ by annealing at 550 K. For Ag, small clusters begin to form at a coverage of ≈0.5 ML.^51^ This aggregation is irreversible, but does not affect the position of the majority adatom species measured by the NIXSW method.

Fig. 1C and D show the minimum-energy configuration for Cu_1_ and Ag_1_ adatoms on the Fe_3_O_4_(001) surface, as determined by HSE-based calculations. The favoured adsorption site for both adatoms is twofold coordinated to surface oxygen atoms between the surface Fe_oct_ rows. Specifically, the adatoms bind to the two oxygen atoms without a subsurface Fe_tet_ (*i.e.* tetrahedrally coordinated Fe) neighbour, where the next Fe_tet_ atom would reside if the bulk structure were continued outward. The calculations predict that the Ag_1_ adatom protrudes further from the surface than the Cu_1_ (Δ*z* = 1.12 Å and Δ*z* = 0.59 Å, respectively), which corresponds remarkably well to the STM apparent heights in the bias range *V*_sample_ = 1–1.5 V.

To quantitatively benchmark the adatom geometry, we performed NIXSW experiments^48^,^49^ at beamline I09, Diamond Light Source. NIXSW exploits the standing wavefield generated by the interference between incident and reflected photon beams at a specific Bragg condition of the substrate. As the incident photon energy is varied near such a condition, the standing wavefield moves relative to the Bragg planes. Since the standing wave also extends beyond the substrate surface, the photoemission intensity from an adatom core level (Ag 3d or Cu 2p, in this case), excited by the X-rays, varies with the photon energy. Maximum (minimum) intensity is observed when the antinode (node) of the standing wavefield coincides with an adatom. An analysis of the NIXSW profile yields two parameters: the coherent position (*P*_*hkl*_) and coherent fraction (*f*_*hkl*_).^48^,^49^ These are, colloquially, the mean position and level of order of the adatom between the Bragg planes. In total we exploited the (004), (113), and (044) reflections (schematically shown in Fig. 1E), occurring at *hν* = 2960, 2450 and 4180 eV respectively. Note that at exact normal incidence, the wavelength of the light will be twice the spacing between the planes. Atomic density maps (*e.g.*Fig. 1F), reconstructed from three reflections using a Fourier expansion described in the ESI,† directly (and unambigiously) identify the three dimensional adsorption site as the surface oxygen bridge site, confirming the site inferred from the STM/calculated data. Here we focus primarily on the (004) data, which specifically probes the vertical positions of the adatoms. Details of the complete NIXSW analysis are included in the ESI.†

Fig. 2 shows the fitted (004) NIXSW profiles following the deposition of 0.4 ML of Cu and Ag at room temperature. The photon energy scale is plotted relative to the Bragg energy, defined by the X-ray reflectivity curve shown in the lower curve. Clearly, the maximum photoemission intensity occurs at significantly different energies for the Ag 3d_5/2_ and Cu 2p_3/2_ data, indicating a significant difference in their position with respect to the Fe_3_O_4_(004) planes. Specifically, *P*_004_ values of 0.71 ± 0.02 (Cu_1_) and 0.96 ± 0.01 (Ag_1_) were obtained, which corresponds to heights of *H*_Cu_ = 0.43 ± 0.03 Å (Cu_1_) and *H*_Ag_ = 0.96 ± 0.03 Å (Ag_1_) above an idealized Fe_oct_O_2_ bulk termination. The corresponding *f*_004_ values are 0.71 ± 0.03 (Cu_1_) and 0.66 ± 0.03 (Ag_1_). Annealing the Fe_3_O_4_(001)/Cu surface to 550 K resulted in a dramatic increase in the coherent fraction to 0.93 ± 0.03, consistent with the conversion of metastable Cu_1_* species (Fig. 1B) into regular Cu_1_ adatoms. The relatively low coherent fraction of Ag_1_ is attributable to clustering.^50^ Since the clusters are likely three dimensional, and thus have an equal occupation of all sites within the projected layer spacings, their contribution to the NIXSW simply lowers the coherent fraction without altering *P*_004_.

**Fig. 2:**
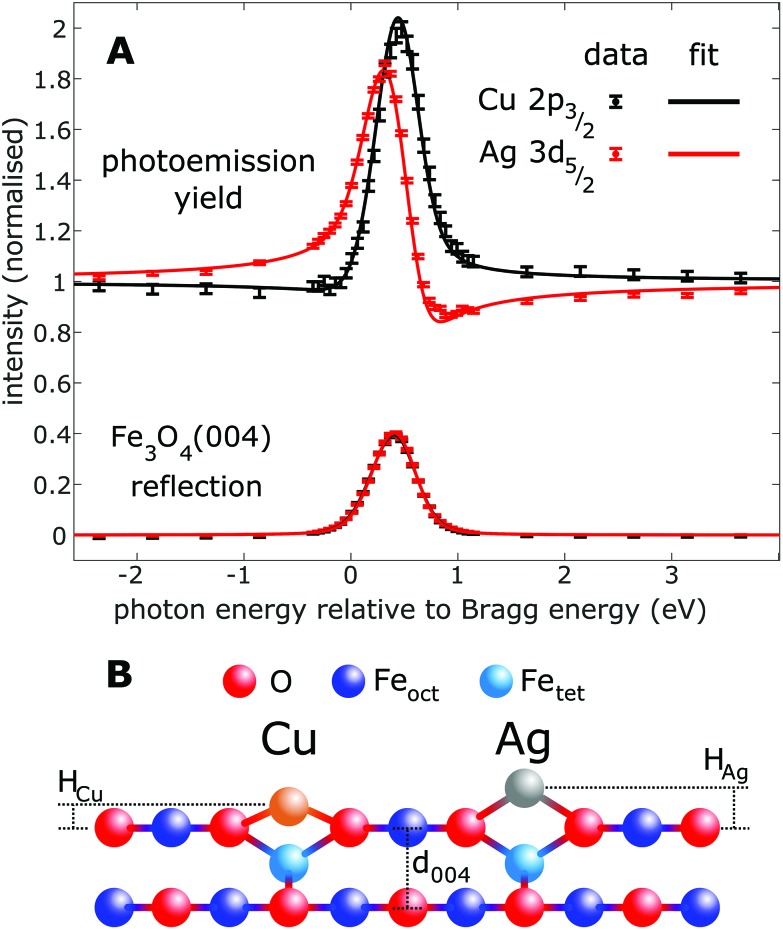
Results of the fitting of the (A) NIXSW data from the (004) reflection of Fe_3_O_4_. (B) Schematic of the apparent heights (*H*_ad_) with respect to a bulk-like terminated Fe_3_O_4_(001) surface. The difference in height is 0.52 ± 0.04 Å, with absolute *H*_Ag_ and *H*_Cu_ values of 0.96 ± 0.03 Å and 0.43 ± 0.03 Å, respectively.

Table 1 shows a selection of the computational results for the Fe_3_O_4_(001)/Cu_1_ and Fe_3_O_4_(001)/Ag_1_ systems. Full details of the various calculations are contained within the ESI,† but briefly, we utilized the Vienna *ab initio* Simulation Package (VASP)^51^,^52^ with the following functionals: PBE,^9^ PBE + U,^9^,^53^,^54^ PBEsol + U,^11^ and HSE.^23^ The surface calculations utilized an asymmetric surface slab with 5 fixed and 2 relaxed layers Fe_oct_O_2_ layers including the SCV reconstruction.^47^ Initially we followed the standard procedure for calculating Fe_3_O_4_ surfaces, using a theoretical lattice parameter obtained by relaxing the bulk unit cell with the relevant functional. PBE + U and HSE overestimate the lattice by 0.75% and 0.18%, respectively, whereas PBE and PBEsol + U underestimate it by 0.01% and 0.61%, respectively. All calculations except the PBE find that the addition of the metal adatom reduces the total magnetic moment from 60*μ*_B_ to 59*μ*_B_ in both cases, indicating a charge state of +1 for the adatoms.

**Table 1:** The adatom geometries obtained from various theoretical approaches and the adatom heights determined in the NIXSW experiment. For PBE + U, both the relaxed and experimental lattice parameter (8.396 Å) were used. Note Δ*z*_ad_ is the height above a relaxed Fe_oct_O_2_ layer, as indicated in Fig. 1C and D, whereas *H*_ad_ is the height above a projected bulk terminations, as described in eqn (1) and indicated in Fig. 2B

Method	HSE	PBE + U	PBE + U	PBE	PBEsol + U	NIXSW
Lattice param. *a* (Å)	8.411	8.459	8.396	8.390	8.345	8.396
*a*–*a*_expt_ (%)	+0.18	+0.75	0	–0.01	–0.61	0
*H*_Ag_ (Å)	0.88	0.78	0.89	0.75	0.64	0.96 ± 0.03
Δ*z*_Ag_ (Å)	1.12	1.00	1.05	0.96	0.85	—
Ag–O bond length (Å)	2.11	2.09	2.09	2.06	2.02	—
*E*_ad_ (eV)	–1.99	–2.19	–1.93	–2.30	–2.63	—

*H*_Cu_ (Å)	0.37	0.33	0.41	0.38	0.31	0.43 ± 0.03
Δ*z*_Cu_ (Å)	0.59	0.53	0.55	0.56	0.50	—
Cu–O bond length (Å)	1.86	1.85	1.84	1.84	1.82	—
*E*_ad_ (eV)	–3.46	–3.76	–3.60	–3.85	–4.17	

To directly compare the theoretical geometry to the NIXSW results, the adatom height must be calculated with respect to the bulk lattice. Thus we must convert the theoretical adatom height with respect to the bottom fixed Fe_oct_O_2_ layer in the DFT slab, *z*_ad_, to *H*_ad_ (ad = Ag, Cu) by:

1 *H*_ad_ = *z*_ad_ – *n·d*_004_,

where *n* = 6 is the number of Fe_oct_O_2_ inter-layer spacings in the DFT slab, and *d*_004_ = 2.099 Å is the bulk (004) layer spacing. Clearly, the HSE results (Table 1) best model the experiment, yielding *H*_ad_ values just below the experimental range. Moreover, the Ag binding energies of 2.0 eV compares well to a recent adsorption calorimetry experiment for Ag on Fe_3_O_4_(111) (2.3 eV).^55^

Interestingly, the predicted charge state (+1 – supported by X-ray photoelectron spectroscopy, see ESI†) and Cu–O and Ag–O bond lengths (1.86 Å and 2.11 Å, respectively) are similar to the bulk compounds Cu_2_O and Ag_2_O (1.8481 ± 0.0004 Å and 2.043 ± 0.002 Å, respectively),^56^ where the cations bind linearly to O^2–^ anions. Thus, the adatom geometry can be understood as the metal adopting its favored bond length to oxygen, with the constraint that the surrounding Fe_3_O_4_ lattice precludes the ideal linear geometry. It is then straightforward to understand why the *H*_ad_ predicted by PBE + U is too low (*H*_Ag_ = 0.78 Å, *H*_Cu_ = 0.33 Å). Although the Cu–O and Ag–O bond lengths are similar to HSE, the overestimation of the lattice parameter leads to a widening of the relevant O–O distance, and the adatoms sink towards the substrate. Simply rescaling the calculation to the experimental lattice makes matters worse, because this also reduces the Ag–O/Cu–O bondlengths, and thus the adatom height. If instead, utilising the PBE + U functional, the substrate lattice parameter is constrained to the experimental value (8.396 Å) at the outset of the calculation, the Ag–O and Cu–O bondlengths are unaffected, and *H*_ad_ values closer to the experiment are obtained. The local structure of the adatoms provides a very good approximation to the HSE results, though it must be noted that constraining the lattice in this way leads to a small expansion in the *z* direction in the relaxed layers. Altering the *U*_(eff)_ parameter did not provide a more accurate modelling of the geometric structure (see ESI/Fig. S5†). Given the importance of the lattice parameter and metal–oxygen bonding in general, one might expect that PBEsol + U, specifically designed to correct for the disfavor of density overlapping of PBE, should perform well. Such calculations do indeed yield a lattice parameter closer to experiment than PBE + U, but the Ag adatom height is dramatically underestimated because the Ag–O bond length is also significantly reduced, therefore constraining the lattice constant to the experimental value resulted in a greater disagreement with experiment. This concomitant overbinding of adsorbates by PBEsol has been observed previously.^21^ A similar overbinding is observed in the PBE calculations (without U), due to highly reduced degree of localization of the Fe 3d electrons. Thus PBE and PBEsol are clearly not a suitable choice for SAC studies.

## Conclusions

In summary, we demonstrate *via* direct NIXSW imaging, that Cu and Ag adatoms occupy a bulk-continuation cation site on the Fe_3_O_4_(001) surface. Furthermore the NIXSW data indicates their height above the surface differs significantly by 0.52 ± 0.04 Å. Successful theoretical modelling of this quantitative experimental result was found to be dependent not only on the choice of functional, but also on the bulk lattice parameter.

Large deviations from the lattice parameter are known to affect calculations of phonon and magnetic properties, but the values obtained here would not normally be considered problematic, especially for adsorption studies. However, the PBE + U functional only obtains quantitative agreement with experiment when the lattice parameter is within 0.2% of the experimental value (8.396 Å). Despite this ability to “shoe-horn” the PBE + U calculations into more accurately modelling the experimental results, it is clear that, by predicting an underbinding of the atoms in the substrate and a relative overbinding between the substrate and the adatom, PBE + U fails at this experimental benchmark. Thus the use of the popular PBE + U functional must be questioned, certainly in its application to metal adatoms supported on magnetite, potentially to single metal adatoms supported on other metal oxides surfaces as well, and possibly even to metal nanoclusters supported on metal oxides in general.

## Conflicts of interest

The authors have no conflicts to declare.

## Supplementary Material

Supplementary information
