# Supplementary material for: Probing the geometry of copper and silver adatoms on magnetite: quantitative experiment versus theory
Source: Nanoscale. 2018 Jan 15;10(5):2226–30. doi: 10.1039/c7nr07319d (PMC5795485; doi:10.1039/c7nr07319d)
Supplement: Supplementary file 1 [file NR-010-C7NR07319D-s001.pdf]

Electronic Supplementary Material (ESI) for Nanoscale.
This journal is © The Royal Society of Chemistry 2017

Probing the geometry of copper and silver adatoms on magnetite: Quantitative experiment versus theory

Matthias Meier[a,b], Zdeněk Jakub[b], Jan Balajka[b], Jan Hulva[b], Roland Bliem[b], Pardeep K. Thakur[c], Tien-Lin Lee[c], Cesare Franchini[a], Michael Schmid[b], Ulrike Diebold[b], Francesco Allegretti[d], David A. Duncan[c]* and Gareth S. Parkinson[b]

[a] University of Vienna, Faculty of Physics and Center for Computational Materials Science, 1090 Vienna, Austria

[b] Institute of Applied Physics, TU Wien, 1040 Vienna, Austria

[c] Diamond Light Source, Harwell Science and Innovation Campus, Didcot, OX11 0QX UK

[d] Physics Department E20, Technical University of Munich, 85748 Garching, Germany.

*email: david.duncan@diamond.ac.uk

1. Experimental details

All experiments were performed on natural Fe_3O_4(001) single crystals prepared under ultrahigh vacuum (UHV – base pressure ~ 3×10^{-10} mbar) conditions by cycles of sputtering (Ar^+, 1 keV, 3 mA emission current, 10 min) and annealing (870 K, 10 min). Every second anneal was performed in an O_2 background (p_{O2} = 10^{-6} mbar) to prevent reduction of the outermost surface layers [1]. Ag and Cu were deposited using an Omicron EFM3 evaporator with the sample held at room temperature, and the deposition rate was monitored by a water-cooled quartz crystal microbalance (QCM). One monolayer (ML) is defined as 1 adatom per Fe_3O_4(001)-(√2×√2)R45° unit cell, or 1.42×10^{14} atoms/cm^2. These metals (Ag and Cu) were selected for this study due to the high adatom densities that can be achieved with minimal cluster formation [2], their resistance to adsorption of the residual gas at room temperature, and because they lie at the extremes of the range of geometries based on preliminary DFT+U calculations. The normal incidence X-ray standing wave (NIXSWR) measurements were performed at a normal incidence geometry with respect to the Bragg plane from which the scattering occurred, thus the photons were only incident on the surface normal for the (004) measurements. The coherent positions and fractions over all three reflections are calculated with respect to a Fe_3O_4 unit cell with the tetrahedral iron atoms at the origin. The apparent height (H_{ad}) of the adatom, with respect to a bulk-like terminated Fe_3O_4(001) surface was calculated from the coherent position of the (004) data, p_{004}, by:

$$H_{ad} = (p_{004} - p_{Fe_{oct}O2}) . d_{004}, \qquad (1)$$

where d_{004} = 2.099 Å is the spacing between (004) scattering planes, and $p_{Fe_{oct}O2} = 0.5$ is the coherent position of the $Fe_{oct}O_2$ plane between the same planes.

2. Computational details

All the theoretical calculations were performed using the Vienna *ab initio* Simulation Package (VASP) [3, 4] using the Projector Augmented Wave (PAW) approach [5, 6] with a basis set cut-off energy of 550 eV. Calculations were initially performed using the Perdew-Burke-Ernzerhof (PBE) [7] exchange correlation functional with an effective on-site Coulomb repulsion term U_{eff} = U-J = 3.6 eV[8]; this is the standard procedure for Fe_3O_4 above the Verwey transition [9-11]. We also tested a variant optimized specifically for solids, PBEsol [12], which accounts for the well-known issue of disfavoured density overlapping present in PBE, using the same U_{eff}. The hybrid functional HSE[13] was investigated with the standard mixing factor 25%, and screening length (0.207^{-1} Å^{-1}). However, due to the expensive computational cost of such calculations the k-meshes were reduced by a factor 2 along each direction. The k-points mesh has been optimized such that it delivers total energy with an accuracy of better than 1 meV and reducing the k-mesh by a factor of 2 at PBE level leads to a change in the total energy of only 1 meV. In all cases, structures were relaxed until forces were smaller than 0.02 eV/Å.

The surface calculations utilized an asymmetric surface slab, resulting in a significantly cheaper calculation, with 9 fixed layers and 4 relaxed layers with the subsurface cation vacancy reconstruction [10]. Due to large size of the unit-cell (~100 atoms) the

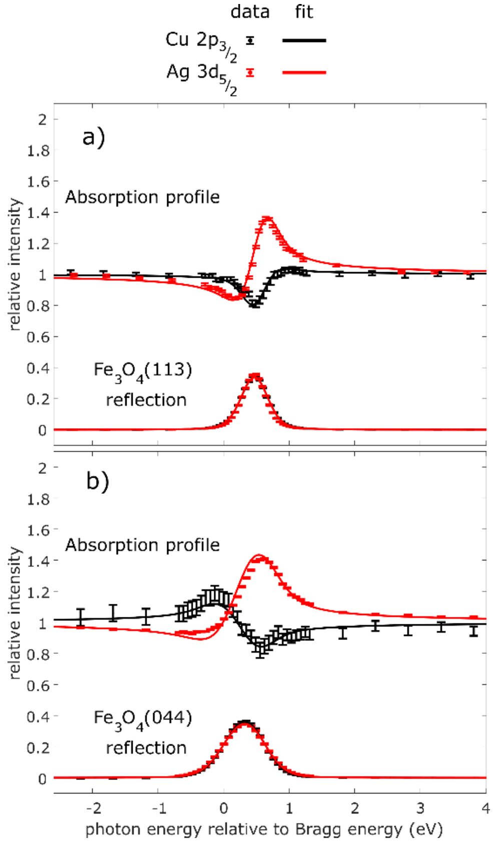

Figure S1: Results of the fitting of the NIXSW data from the a) (113) and b) (044) reflection of Fe3O4. The absorption profiles were obtained from the photoemission yield of Cu $2p_{3/2}$ and Ag $3d_{5/2}$ core levels.

Table S1: Measured coherent fractions and positions for Cu and Ag adatoms adsorbed on the Fe_3O_4(001) surface, acquired from the Cu 2p $^3/_2$ and Ag 3d $^5/_2$ energy levels, respectively. Quoted uncertainties are the error in the fit at two standard deviations. The coherent positions are based on the origin of the Fe3O4 lattice coinciding on a tetrahedral iron cation, as indicated in Fig. 1e in the manuscript.

	f_{004}	p_{004}	f_{044}	p_{044}	f_{113}	p_{113}
0.4 ML Cu	0.71±0.03	0.71±0.02	0.89±0.03	0.71±0.01	0.71±0.02	0.68±0.01
0.4 ML Ag	0.66±0.03	0.96±0.01	0.60±0.03	0.95±0.02	0.56±0.02	0.82±0.01
0.4 ML Cu (annealed)	0.93±0.05	0.72±0.02	1.00±0.06	0.69±0.02	--	--
Fe_{tet} (ideal)	1.00	1.00	1.00	1.00	0.707	0.875

adoption of a symmetric setup would be computationally prohibitive for HSE-type calculations. The vacuum "thickness" (separation between adjacent supercells perpendicular to the surface) was set to 14 Å, and the k-mesh 5 × 5 × 1. Initially standard procedure was followed: the surface slabs were based on a theoretical lattice parameter obtained by relaxing the bulk unit cell with the relevant functional (Fd$\bar{3}$m structure above the Verwey transition with a Γ-centered k-mesh of 5 × 5 × 5). PBE+U and HSE overestimate the lattice by 0.75% and 0.18 %, respectively, whereas PBEsol+U underestimates by 0.61%. Large deviations from the lattice parameter are known to affect calculations of phonon and magnetic properties, but the values obtained here would not be considered problematic, especially for calculations of adsorption geometries and energies. As shown in the main text however, agreement with experiment is only obtained when a lattice parameter within 0.2% of the experimental value (8.396 Å) is used. Furthermore, under such conditions the PBE+U and HSE functionals produce similar results.

The adsorption energy of the metal adatom on the surface E_{ad} is defined by:

$$E_{ad} = E_{ad+surf} - E_{surf} - E_{isolated}, \quad (2)$$

i.e. the adsorption energy is the difference between the total energy of the adatom surface slab and the combined total energy of the clean Fe_3O_4(001) surface and an isolated metal atom in vacuum.

3. 0.4 ML Cu after annealing

As discussed in the main text, and shown in Table S1, after annealing the Fe_3O_4(001)/Cu layer to approximately 550 K a significant increase in the coherent fraction in the Cu 2p XSW was observed. This is attributed to the removal of the metastable Cu adatom site observed in the STM (Cu_1* in Fig. 1b). No difference is observed in the coherent position, suggesting one of the following three scenarios: (i) the alternative Cu_1 site has a poorly defined adsorption height, centered around that of the regular Cu_1 site; (ii) it has an adsorption height exactly 0.5 layer spacings of the (004) above or below that of the regular Cu_1 site (1.05 Å), which would result in an anti-phase absorption profile to the regular Cu_1 site; or (iii) has a completely random adsorption height over the (004) spacing (2.1 Å).

4. Direct imaging of adatom adsorption site

The coherent fraction, f_H, and coherent position, P_H, introduced in the main text with a colloquial definition, can be more specifically interpreted as the amplitude and phase (respectively) of the Hth-order Fourier component, $\mathcal{F}_H$, of the element specific geometrical structure factor, $\mathcal{F}$. [14-16] By monitoring the photoemission yield across several symmetrically inequivalent reflections of the substrate, the real space atomic density, $\rho_{(x,y,z)}$, can be directly imaged by the Fourier expansion: [17, 18]

$$\rho_{(x,y,z)} \propto \sum f_H \cos(2\pi(P_H - [hx + ky + lz])). \quad (3)$$

As mentioned in the text, the NIXSW experiments presented here exploited the (004), (113) and (044) reflections (schematically shown in Fig. 1E in the main manuscript) of the Fe_3O_4(001) single crystal. The latter two reflections are equivalent to an additional three reflections each. Specifically, the (113) reflection is symmetrically equivalent to: $(\bar{1}13)$, $(1\bar{1}3)$ and $(\bar{1}\bar{1}3)$; and the (044) is symmetrically equivalent to: (404), $(\bar{4}04)$ and $(0\bar{4}4)$. Resulting in a total of 9 Fourier components for the Fourier expansion.

Figure 1f shows a two dimensional slice of the atomic density map, obtained from the expansion of eqn. (3) for all three acquired, and symmetrically equivalent, reflections from the Cu $2p\ _{3/2}$ core level and is reproduced without the overlayed atoms in Fig. S3a. The corresponding map, for the results from the Ag $3d\ _{5/2}$ core level, is shown in Fig. S3b. The Fe_3O_4(001) surface has four possible terminations, which are the four Fe_{oct}O

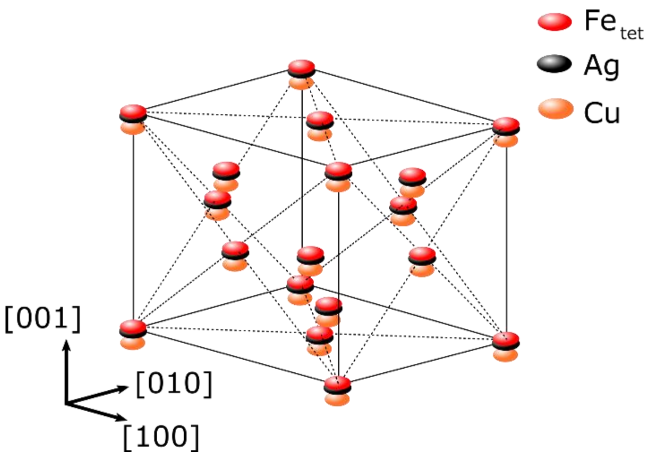

Figure S2: Three dimensional isosurface map of the experimental atomic density of the Ag and Cu adatoms on the Fe_3O_4(001) surface from the expansion of equation 3, compared to a comparable map of an ideal set of tetrahedral Fe atoms. The isosurface is taken at 80% of maximum density in order to remove the artifacts, as shown in Fig. 1f and S3. The solid black lines indicate the Fe_3O_4 unit cell, whereas the dashed lines indicate the primary diagonal of each lattice face.

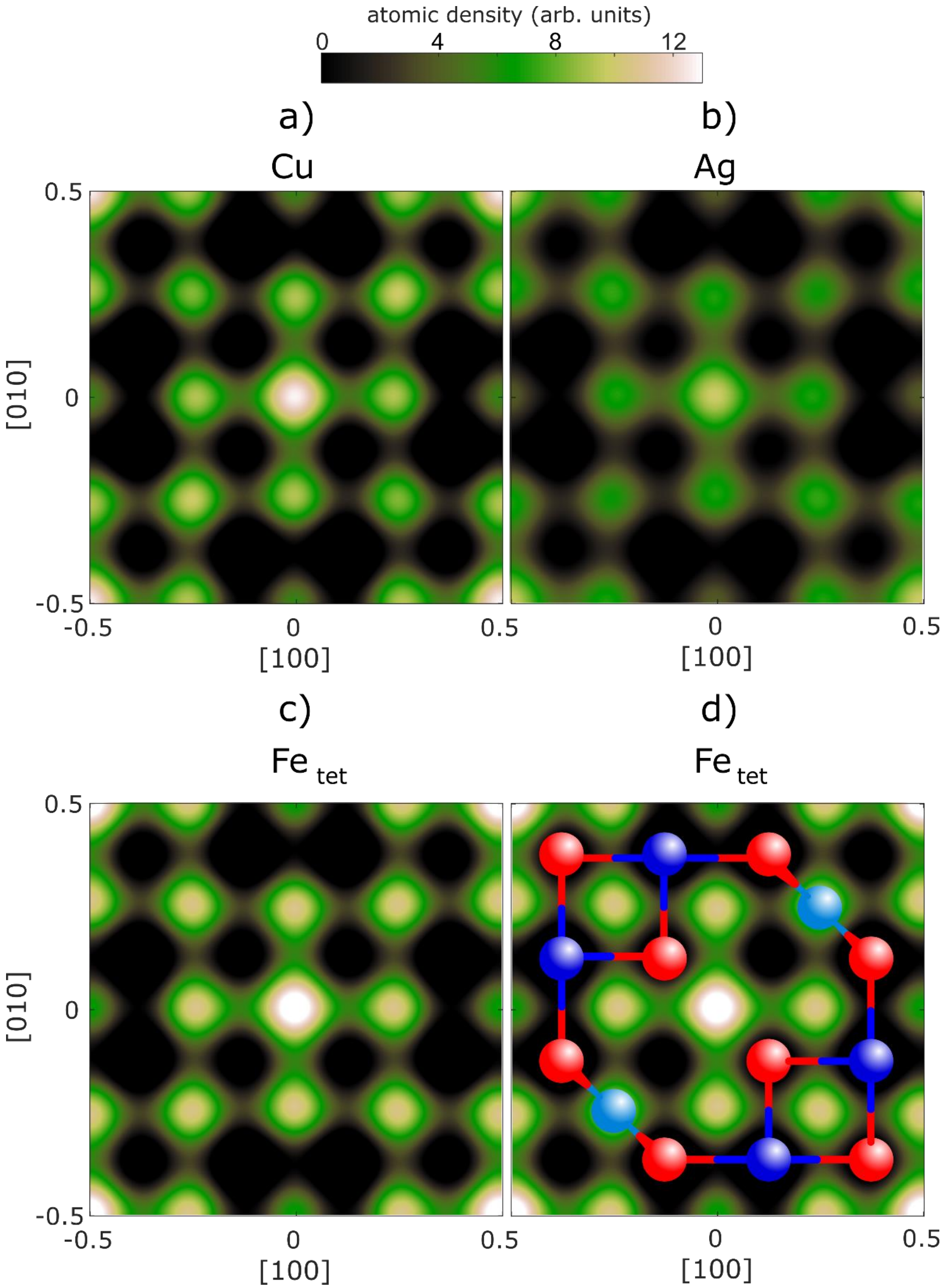

Figure S3: Two dimensional (2D) slice of the experimental atomic density map obtained from the expansion of eq. (3) for (a) Cu, (b) and Ag adatoms in comparison to a similar such map – (c) and (d) – obtained from the calculated coherent fractions and positions (shown in Table S1) for Fe_{tet} in bulk sites. Panel (d) has a ball and stick schematic, with the same colour scheme as Fig. 2, of the first $Fe_{oct}O$ and Fe_{tet} layers below the Fe_{tet} layer that this two dimensional slice is transecting. In all cases the slice is taken at a position in the [001] direction where the maximum atomic density is found and is specifically, as a fraction of the (001) spacing, (a) 0.93, (b) 0.99 and (c-d) 1.00. Note that the correct site, for the Fe_{tet} in the layer of this 2D slice is at (0,0), (±0.5, ±0.5) and (±0.5, ∓0.5).

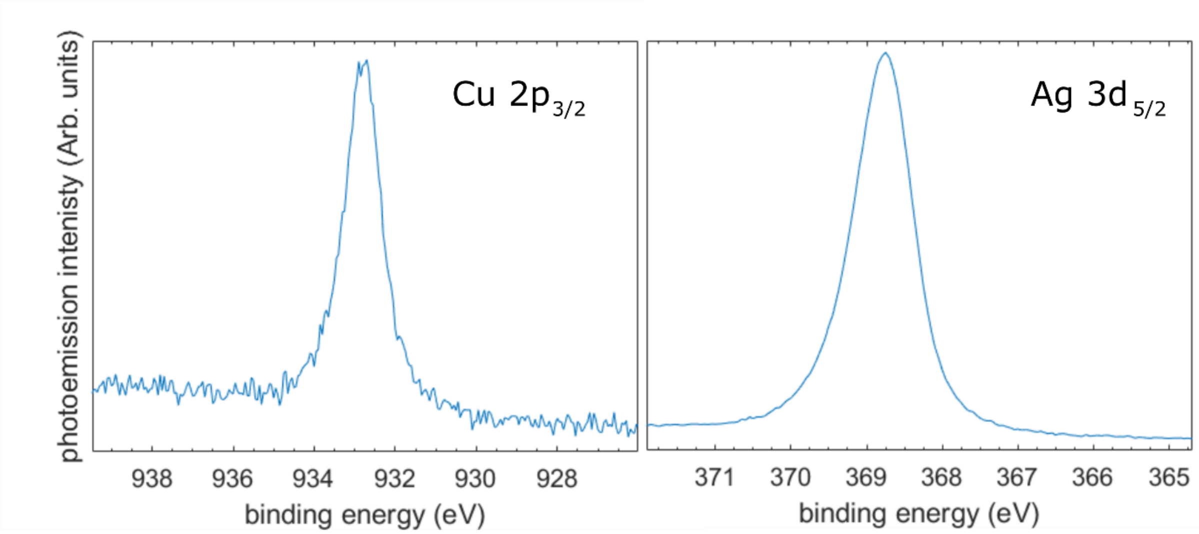

Figure S4: Cu $2p_{3/2}$ and Ag $3d_{5/2}$ XP spectra from the Fe_3O_4(001)/Cu_1 and Fe_3O_4(001)/Ag_1 systems. The binding energy of the maximum of each peak is 932.8 and 368.8 eV, respectively.

layers in the bulk unit cell. For simplicity, only the results for adsorption of the adatoms above one of these layers are presented as 2D maps. The slice corresponds to a plane that is parallel to the Fe_3O_4(001) surface, at the determined height of the respective adatom, obtained directly from the (004) reflection. The atomic density maps, display two global maxima (at the centre and the corners) as well as multiple local maxima. These local maxima are assigned to artifacts originating from utilising a finite set of crystallographic planes resulting in a truncation error in the Fourier expansion, and can be discounted as the global maxima are 40% more intense than these local ones, and, as discussed below, a theoretical reconstruction of the Fe_{tet} exhibits identical artifacts. Thus the Cu and Ag adatoms are assumed to occupy only the sites corresponding to the global maxima, which are all located in sites bridging two oxygen atoms. In the bulk-terminated Fe_3O_4(001) surface there are two such sites, specifically one site with a Fe_{tet} atom directly below and one site without; the global maxima, and thus the adatoms, lie in the latter. However, due to the (√2×√2)R45° reconstruction of the surface half of these sites contain an interstitial Fe_{tet} directly below it [10]. As the NIXSW determines the adsorption site with respect to the bulk lattice, it is insensitive to the surface reconstruction and cannot discriminate between these two different surface sites. It is likely, though, that the adatom sits in the site without any Fe atom directly below it, as is favoured by our DFT-based calculations.

As mentioned above, the Fe_3O_4(001) surface has four possible terminations, each a symmetrically identical $Fe_{oct}O$ layer. These four layers are related by a 90° rotation and a translation of magnitude $\sqrt{2} \times 0.25a$ along the $[1\bar{1}0]$ direction, where a is the bulk lattice parameter. All four terminations are equivalent, therefore all will be present on the sample surface and the adatoms will occupy symmetrically identical sites at all four terminations. In Fig. 1 the atomic density map, obtained from the expansion of eqn. (3), is shown for a single one of these terminations. Figure S2 shows a three dimensional isosurface map, of the atomic density, over the whole unit cell for Ag and Cu. For comparison an atomic density map was calculated from the theoretical coherent fractions and positions for a tetrahedral Fe atom (shown in Table S1) and included in Figure S2. The two dimensional slice of the atomic density map that is comparable to Fig. 1f for the ideal tetrahedral Fe atoms and the Ag adatom, is shown in Fig. S3b-d. Figure S3a shows Fig. 1f without the overlayed atomic positions for clarity.

5. Cu $2p_{3/2}$ and Ag $3d_{5/2}$ X-ray photoelectron spectroscopy

Representative Cu $2p_{3/2}$ and Ag $3d_{5/2}$ X-ray photoelectron spectra are shown in Figure S4. The Cu $2p_{3/2}$ spectra were measured at a photon energy of 1100 eV, and the Ag $3d_{5/2}$ spectra at 600 eV. In both cases the absolute binding energy was calibrated against the Fermi energy acquired at the same photon energy. The Cu $2p\ {}^{3}/_{2}$ and Ag $3d\ {}^{5}/_{2}$ XPS measurements exhibit a binding energy of 932.8 eV and 368.8 eV, respectively, consistent with a 1+ oxidation state [19, 20] (Note that the absolute binding energies of Ag oxidation states are controversial in the literature[20], however 368.8 eV is a somewhat higher binding energy than the well-defined Ag $3d\ {}^{5}/_{2}$ binding energy of bulk metallic silver at 368.3 eV[21-23]).

6. PBE+U calculations: $U_{(eff)}$

The PBE+U calculations presented in the main body of the text utilize a $U_{(eff)}$ of 3.61 eV, which was chosen because it accurately models the electronic structure of the bulk magnetite[24, 25]. Specifically a $U_{(eff)}$ of 3.61 eV reproduces the magnetic moments well, predicts a small gap in the majority spin contribution resulting in a bulk semi-metal and reproduces the charge disproportion when performing a Bader charge analysis. However, as discussed in the main text, this $U_{(eff)}$ fails to model the adsorption structure of the metal adatoms accurately. This raises the question of whether there is a $U_{(eff)}$ that would model the structure properly, at the expense of modelling the electronic properties improperly. To this end we ran a series of calculations over a range of $U_{(eff)}$ values between 2 and 5 eV. These calculations were performed with a basis set cut-off energy of 350 eV, with a k-mesh of 2x2x1. From these calculations the resulting H_{Ag}, H_{Cu} (as described in eqn. (1) in the main text) and lattice parameter (a) were compared against the experimental values, shown in Fig. S5. When the $U_{(eff)}$ values are set significantly lower than 3.61 eV then the lattice parameter tends towards the experimental value; above

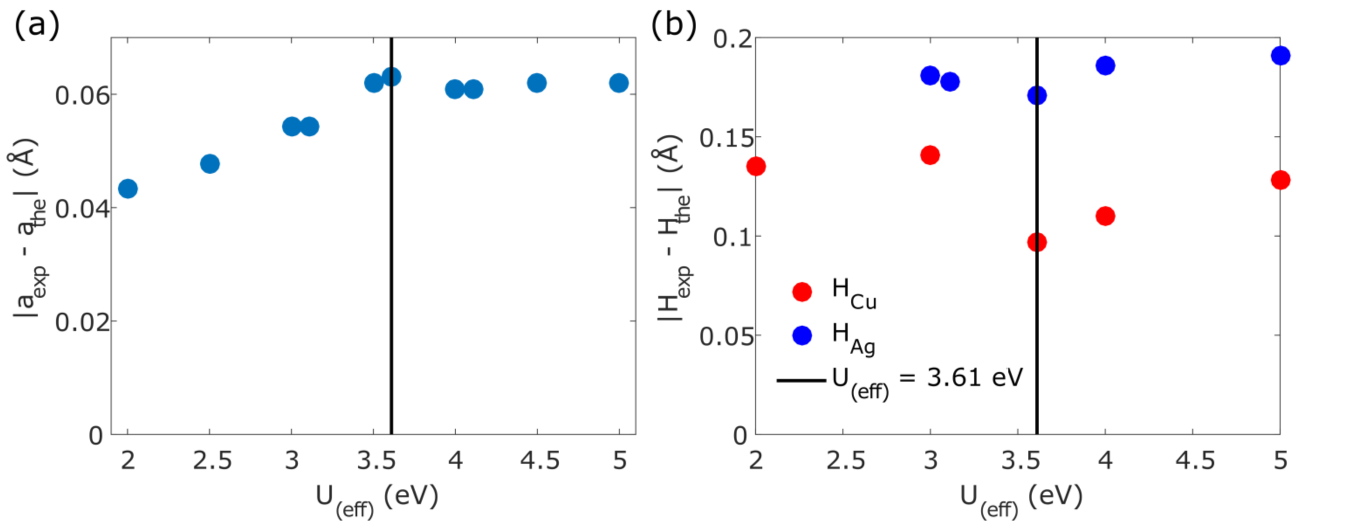

Figure S5: Difference between the experimental and theoretical (a) lattice constants and (b) adatom adsorption heights as a function of $U_{(eff)}$. The results using a $U_{(eff)}$ of 3.61 eV are highlighted by a black line.

3.61 eV the lattice parameter plateaus. A $U_{(eff)}$ value of 3.61 eV corresponds to the minimum difference, between experiment and theory, in the adsorption height of the copper adatom, but varying $U_{(eff)}$ had little to no effect on the adsorption height of the Ag adatom. Note that in all cases the adsorption height of both adatoms is underestimated and the lattice parameter is overestimated. Thus is not possible to "fudge" the correct result by variation of this parameter.

References

1. G. S. Parkinson, Z. Novotný, P. Jacobson, M. Schmid and U. Diebold, *Surface Science*, 2011, **605**, L42-L45.
2. R. Bliem, R. Kosak, L. Perneczky, Z. Novotny, O. Gamba, D. Fobes, Z. Mao, M. Schmid, P. Blaha, U. Diebold and G. S. Parkinson, *ACS Nano*, 2014, **8**, 7531-7537.
3. G. Kresse and J. Hafner, *Physical Review B*, 1993, **48**, 13115-13118.
4. G. Kresse and J. Furthmüller, *Computational Materials Science*, 1996, **6**, 15-50.
5. P. E. Blöchl, *Physical Review B*, 1994, **50**, 17953-17979.
6. G. Kresse and D. Joubert, *Physical Review B*, 1999, **59**, 1758-1775.
7. J. P. Perdew, K. Burke and M. Ernzerhof, *Physical Review Letters*, 1996, **77**, 3865-3868.
8. S. L. Dudarev, G. A. Botton, S. Y. Savrasov, C. J. Humphreys and A. P. Sutton, *Physical Review B*, 1998, **57**, 1505-1509.
9. Z. Łodziana, *Physical Review Letters*, 2007, **99**, 206402.
10. R. Bliem, E. McDermott, P. Ferstl, M. Setvin, O. Gamba, J. Pavelec, M. A. Schneider, M. Schmid, U. Diebold, P. Blaha, L. Hammer and G. S. Parkinson, *Science*, 2014, **346**, 1215-1218.
11. Z. Novotny, N. Mulakaluri, Z. Edes, M. Schmid, R. Pentcheva, U. Diebold and G. S. Parkinson, *Physical Review B*, 2013, **87**, 195410.
12. J. P. Perdew, A. Ruzsinszky, G. I. Csonka, O. A. Vydrov, G. E. Scuseria, L. A. Constantin, X. Zhou and K. Burke, *Physical Review Letters*, 2008, **100**, 136406.
13. J. Heyd, G. E. Scuseria and M. Ernzerhof, *The Journal of Chemical Physics*, 2003, **118**, 8207-8215.
14. N. Hertel, G. Materlik and J. Zegenhagen, *Zeitschrift für Physik B Condensed Matter*, 1985, **58**, 199-204.
15. M. J. Bedzyk and G. Materlik, *Physical Review B*, 1985, **31**, 4110-4112.
16. M. J. Bedzyk and P. Fenter, in *The X-Ray Standing Wave Technique*, WORLD SCIENTIFIC, 2011, vol. Volume 7, pp. 289-302.
17. L. Cheng, P. Fenter, M. J. Bedzyk and N. C. Sturchio, *Physical Review Letters*, 2003, **90**, 255503.
18. M. J. Bedzyk and L. Cheng, *Reviews in Mineralogy and Geochemistry*, 2002, **49**, 221.
19. J. Li, Z. Mei, L. Liu, H. Liang, A. Azarov, A. Kuznetsov, Y. Liu, A. Ji, Q. Meng and X. Du, *Scientific Reports*, 2014, **4**, 7240.
20. A. M. Ferraria, A. P. Carapeto and A. M. Botelho do Rego, *Vacuum*, 2012, **86**, 1988-1991.
21. M. P. Seah, G. C. Smith and M. T. Anthony, *Surface and Interface Analysis*, 1990, **15**, 293-308.
22. M. Romand, M. Roubin and J. P. Deloume, *Journal of Electron Spectroscopy and Related Phenomena*, 1978, **13**, 229-242.
23. M. T. Anthony and M. P. Seah, *Surface and Interface Analysis*, 1984, **6**, 95-106.
24. A. Kiejna, T. Ossowski and T. Pabisiak, *Physical Review B*, 2012, **85**, 125414.
25. I. Bernal-Villamil and S. Gallego, *Journal of Physics: Condensed Matter*, 2015, **27**, 293202.
